# Cognitive–Psychological Characteristics Influencing Weight Loss in Severe Obesity

**DOI:** 10.3390/nu17030581

**Published:** 2025-02-05

**Authors:** Simona Calugi, Gianmatteo Cattaneo, Mirko Chimini, Anna Dalle Grave, Alexandra Balosin, Giulia Bozzato, Riccardo Dalle Grave

**Affiliations:** Department of Eating and Weight Disorders, Villa Garda Hospital, 37016 Verona, Italy; si.calugi@gmail.com (S.C.); gianmatteocattaneo@gmail.com (G.C.); mirko.chimini1@gmail.com (M.C.); annadallegrave@gmail.com (A.D.G.); alexandra.balosin@gmail.com (A.B.); gbozzato3@gmail.com (G.B.)

**Keywords:** obesity, treatment, outcome, cognitions, predictors, weight stigma, binge eating, eating disorder

## Abstract

**Background/Objectives**: Cognitive and psychological factors, such as eating disorder psychopathology, irrational food beliefs, and internalized weight stigma, have not been sufficiently explored in the context of obesity treatment. This study evaluated the role of these variables as predictors of weight loss in patients with severe obesity following a brief intensive cognitive–behavioral therapy for obesity (CBT-OB) program. **Methods**: A total of 400 patients (mean BMI: 41.9 kg/m^2^; mean age: 55.9 years) participated in a 21-day residential CBT-OB intervention, followed by a 12-month follow-up assessment. The Eating Disorder Examination Questionnaire, Weight Bias Internalization Scale, and Irrational Food Beliefs Scale were administered at admission and discharge. Body weight was also assessed at the 12-month follow-up. **Results**: Of the participants, 371 patients (92.2%) completed the intensive CBT-OB program, and 310 (81.1%) attended the follow-up. On average, completers achieved 9% weight loss at follow-up, accompanied by a significant reduction in binge-eating episodes. Cognitive factors, including lower baseline eating concern, higher baseline weight concern, and greater improvement in irrational beliefs (specifically self-deception about eating and weight control), significantly predicted weight loss at 12 months. However, internalized weight stigma did not predict weight loss in this cohort. **Conclusions**: This study underscores the importance of targeting specific cognitive factors in obesity treatment to enhance long-term outcomes. Addressing irrational food beliefs and promoting flexible dietary restraint may improve weight loss and maintenance in individuals with severe obesity. Further research is warranted to refine cognitive–behavioral interventions for personalized obesity management strategies.

## 1. Introduction

Current obesity treatment strategies typically involve lifestyle modification programs, including diet, exercise interventions, pharmacotherapy, and bariatric surgery [1]. However, the efficacy of these approaches varies widely among individuals, highlighting the need for a more nuanced understanding of the factors that contribute to successful weight management [2,3,4].

In real-world settings, where control over diet and physical activity is less stringent, weight loss outcomes can vary significantly, even among individuals following the same dietary regimen [5]. This variability is often attributed to genetic, biological, or metabolic differences among individuals, leading to increased interest in personalized or precision nutrition [5,6]. Intervention studies have yet to confirm the predictive value of these factors [7].

Beyond biological and environmental influences, cognitive mechanisms may play a critical role in determining the success of weight management strategies [8]. Cognitive factors, however, have been largely overlooked in traditional weight-loss programs, which may in part contribute to their limited long-term effectiveness. Emerging evidence suggests that specific cognitive factors, such as weight-loss expectations, motivation, dietary restraint, and disinhibition, are associated with varying degrees of success in obesity treatment [9]. A large Italian study of 1944 patients in real-world settings found that higher weight-loss expectations and appearance-based motivations were associated with treatment discontinuation. At the same time, increased dietary restraint and reduced disinhibition were linked to greater weight loss. Long-term maintenance was associated with satisfaction with results and confidence in self-managed weight loss [10,11]. These data highlight the importance of considering these factors in obesity management.

Despite these findings, other cognitive and psychological factors, such as eating disorder psychopathology, irrational food beliefs, and internalized weight stigma (IWS), have not been sufficiently explored in the context of obesity treatment. These variables are particularly relevant due to their influence on cognitive distortions, emotional regulation, and problematic behaviors associated with weight control.

Eating disorder psychopathology includes dietary restraint and eating, shape, and weight concerns. The main studies have focused on dietary restraint and weight loss, and a recent narrative review investigating this topic highlighted that an increase in flexible restraint and a decrease in rigid restraint is related to greater weight loss [12]. Moreover, a high rate of weight and shape concern when starting behavioral weight loss has been associated with poorer weight loss [13,14], but this finding was not completely confirmed in a more recent study [15].

Cognitive distortions, such as irrational food beliefs, may also hinder weight control efforts, although their role in weight loss remains under-researched [16]. Some studies have also suggested that IWS may predict less weight loss and maintenance, although the findings differ by race and gender [17,18]. However, its impact on behavioral weight-loss treatment and long-term outcomes remains under-researched, with existing studies offering mixed results [19].

Given the limited research on cognitive factors influencing weight loss, this study aims to assess how changes in cognitive eating-related psychopathology, irrational food beliefs, and internalized weight stigma after a brief, intensive cognitive–behavioral treatment program for obesity (CBT-OB) predict weight loss at a 12-month follow-up in patients with severe obesity [20]. By examining these factors, the study seeks to assess the role of some cognitive factors in influencing weight loss in patients with obesity seeking treatment, enhance the understanding of cognitive influences on weight loss, and contribute to the development of more effective, personalized obesity treatment strategies.

## 2. Materials and Methods

### 2.1. Intensive CBT-OB

The primary goals of CBT-OB are to help patients: (i) achieve, accept, and sustain a healthy level of weight loss (5–10% of their initial body weight) [13]; (ii) adopt and maintain a lifestyle conducive to weight control; and (iii) develop a stable “weight-control mindset”. CBT-OB therapists foster a collaborative working relationship, emphasizing teamwork between the therapist and patient(s).

The treatment integrates dietary and exercise recommendations with behavioral and cognitive therapeutic techniques. In addition to strategies commonly used in traditional behavioral obesity treatments, such as self-monitoring, goal setting, stimulus control, contingency management, behavioral substitution, social support, problem-solving, and relapse prevention [21], CBT-OB incorporates cognitive strategies adapted from enhanced CBT (CBT-E) for eating disorders [22]. These include engaging patients in prioritizing treatment, taking an active role in habit change, structuring session agendas, real-time self-monitoring, strategic homework assignments, establishing regular eating patterns, and promptly addressing setbacks. Elements from Cooper et al.’s CBT [23] are also included, such as distinguishing between weight loss and weight maintenance, addressing unrealistic weight goals, primary motivations, and body image concerns during the weight loss phase.

CBT-OB also incorporates the following key components: (i) actively involving, with patient consent, significant others to create a supportive environment for dietary and physical activity changes; (ii) providing a structured meal plan based on food exchange lists; (iii) training patients to assess energy expenditure and develop both an active lifestyle and improved physical fitness; (iv) collaboratively developing a personalized formulation of processes hindering weight loss; (v) encouraging patients to complete the Weight-Loss Obstacles Questionnaire weekly to identify behavioral and cognitive barriers to weight loss; (vi) extending the maintenance phase (48 weeks vs. 24 weeks); and (vii) offering two intensive care steps—day-hospital and residential CBT-OB—for patients with severe and disabling obesity [20,21].

The intensive CBT-OB program is a residential specialized 21-day treatment for severe obesity. It is based on a personalized CBT-OB framework encompassing three key intervention areas: (i) Dietary Intervention: A low-calorie diet consisting of 25% protein, 20% fat, and 45% carbohydrates, designed to induce a daily energy deficit of 500 kcal. Daily multivitamin supplements were also provided; (ii) Physical Activity Program: This included 30 min of indoor cycling daily, supplemented by two 45-min calisthenics sessions per week; and (iii) Cognitive–Behavioral Therapy: The CBT component comprised 15 group sessions covering:

- Self-monitoring: Focused on tracking food intake, physical activity, and body weight.
- Stimulus Control: Techniques to minimize food-related stimuli in the home environment.
- Problem-Solving: Proactive approaches to managing events influencing eating habits and mood.
- Cognitive Restructuring: Addressing and modifying dysfunctional thoughts that impede weight loss to foster a sustainable weight control mindset.
- Relapse Prevention: Developing skills to prevent relapse.

Patients were provided with a manual outlining these strategies [24]. Additionally, significant others of the patients participated in two group sessions aimed at educating them about obesity and creating a supportive home environment conducive to weight management.

Following the intensive residential program, patients were routinely offered the opportunity to engage in a 12-month outpatient program.

### 2.2. Participants

The study included 461 patients with obesity who were consecutively admitted to the residential rehabilitation program at the Department of Eating and Weight Disorders of Villa Garda Hospital between January and December 2022. Participants were referred by general practitioners across Italy. The indication for residential treatment was determined by a global score > 25 on the Comprehensive Appropriateness Scale for the Care of Obesity in Rehabilitation (CASCO-R) [25]. The inclusion criteria for the present study were: (1) age between 18 and 80 years and (2) BMI ≥ 30.0. Excluded patients included (1) those with a diagnosis of bulimia nervosa and bulimia nervosa of low frequency and/or limited duration, as assessed via the Eating Disorder Examination (EDE) interview [26,27], and (2) those who did not complete the self-report questionnaires. Of the 461 eligible patients, 17 (3.7%) were excluded due to meeting DSM-5 diagnostic criteria for an eating disorder. Specifically, these patients included patients with bulimia nervosa (*n* = 4) and low-frequency and/or limited-duration bulimia nervosa (*n* = 13).

**Figure 1:**
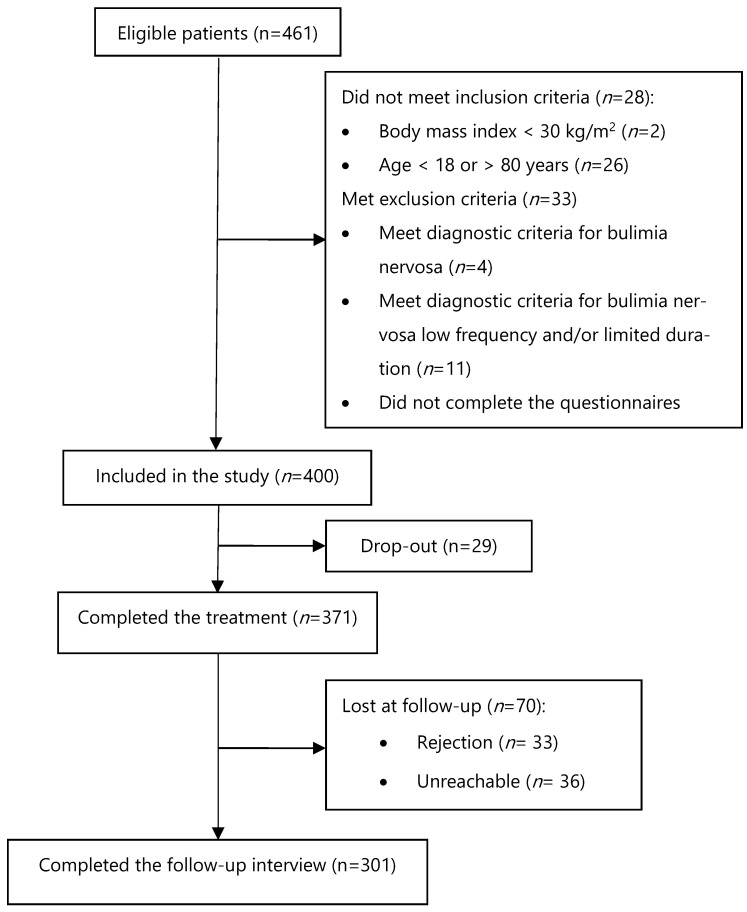
Flow chart.

The final study cohort consisted of 400 patients with obesity (Figure 1).

All participants provided informed written consent for their clinical data to be collected and anonymized within a service-level research setting. The study was approved by the GHG Intuitional Review Board (Protocol Code: 0021GHCIRB).

### 2.3. Assessment

Data collection included a Case Report Form, body weight and height measurements, assessment of residential treatment appropriateness, evaluation of eating disorder psychopathology and behaviors, and analysis of general psychiatric features. Baseline assessments were conducted on the first day of admission. Body weight, as well as eating disorder psychopathology and behaviors, were reassessed at discharge. A follow-up interview and self-reported body weight data were collected 12 months post-discharge.

#### 2.3.1. Case Report Form

The Case Report Form was used to document demographic data, weight, and diet history. Physicians completed it during direct patient interviews on the first day of admission.

#### 2.3.2. Body Weight and Height

Body weight was measured using a calibrated Seca digital wheelchair scale (Model 664, Hamburg, Germany) at baseline, with patients wearing light clothing and no shoes. Height was measured at baseline using a Wunder wall-mounted stadiometer (Model 00051A, Milan, Italy). BMI was calculated using the standard formula: weight (kg)/height (m^2^). Body weight and height were recorded at both admission and discharge, with self-reported weight provided at the 12-month follow-up.

#### 2.3.3. Appropriateness of Residential Treatment

Appropriateness was evaluated using the CASCO-R scale, developed by the Italian Society of Obesity (SIO) and the Italian Society for the Study of Eating Disorders (SISDCA) to determine the suitability of different care settings in Italy (i.e., residential rehabilitation, intensive outpatient rehabilitation, or outpatient treatment). The CASCO-R includes four sections: (i) BMI and waist circumference, (ii) comorbidities associated with obesity, (iii) risk factors increasing obesity-related morbidity, and (iv) previous hospitalization for metabolic/nutritional rehabilitation. Scores are assigned to each item, with a global score >25 indicating severe obesity warranting residential treatment. The CASCO-R has demonstrated strong correlations with overall workload and adverse clinical events, along with excellent internal validity and Test–retest reliability [25].

#### 2.3.4. Eating Disorder Psychopathology and Behaviors

Eating disorder psychopathology and behaviors were assessed at baseline and discharge using the validated Italian version of the Eating Disorder Examination Questionnaire (EDE-Q) [27,28]. The EDE-Q is a self-report tool measuring eating disorder symptoms over the past 28 days, including binge eating, self-induced vomiting, laxative misuse, and excessive exercise. It includes four subscales—Restraint, Eating Concern, Weight Concern, and Shape Concern—and a global score. Items are rated on a 7-point scale (0–6). The Italian EDE-Q version has excellent criterion validity and high test–retest reliability (r = 0.80), with a global score inter-rater reliability of rho = 0.97 [28]. In this study, the EDE-Q global score had a Cronbach’s alpha of 0.83.

#### 2.3.5. Irrational Food Beliefs

Cognitive distortions and inappropriate attitudes or beliefs about food were assessed using the Irrational Food Beliefs Scale (IFBS) [16]. Subjects respond on a 4-point Likert scale ranging from “strongly disagree” to “strongly agree”. IFBS consists of two subscales, one comprising 41 items that investigate irrational beliefs associated with food (irrational subscale), while the other 16 items investigate rational beliefs (rational subscale), making a total of 57 items. The Italian version of the irrational subscale (used in this study) included six factors: ‘self-deception on eating and weight control’, ‘beliefs about eating and emotion regulation’, ‘low tolerance for eating control’, ‘beliefs about eating and hedonic pleasure’, ‘beliefs about dieting’, and ‘all-or-nothing thinking about eating‘ [29].

#### 2.3.6. Internalized Weight Stigma

The Weight Bias Internalization Scale (WBIS) is a self-report questionnaire to measure how individuals internalize negative weight-related stereotypes and attitudes [30]. It assesses personal acceptance of weight stigma and the impact of such stigma on one’s self-esteem and self-worth. The WBIS includes items rated on a Likert scale, where respondents indicate their level of agreement with statements reflecting internalized weight bias. Higher scores on the WBIS indicate greater internalization of weight bias. This scale has been validated in Italian and is widely used in research on weight stigma and its psychological effects [31].

#### 2.3.7. 12-Month Follow-Up Interview

At 12 months post-discharge, all patients were contacted for a telephone interview conducted by researchers not involved in their treatment. This interview, a routine component of the unit’s assessment protocol, collected information on current body weight, lowest post-treatment weight, satisfaction with weight loss, and post-discharge treatment adherence. It also assessed general health status, mood, and binge-eating episodes over the past 28 days. Binge-eating episodes were categorized as follows: 1 = less than 1 episode per week; 2 = 1 episode per week; 3 = 2–3 episodes per week; 4 = 4–7 episodes per week; 5 = 8–13 episodes per week; 6 = 14 or more episodes per week.

### 2.4. Statistical Analysis

Descriptive statistics are presented as means and standard deviations for continuous variables and as percentages for categorical variables.

The *T*-test for paired samples was used to compare clinical variables at admission and at discharge.

Changes in body weight from baseline to the 12-month follow-up were analyzed using repeated measure (RM) ANOVA. RM ANOVA was performed for both completers and intention-to-treat analyses, with missing body weight data at follow-up handled using a multiple imputation procedure via the fully conditional specification method.

Forward stepwise logistic and linear regression analysis was used to evaluate potential cognitive–psychological predictors of treatment. The proportion of patients who lost 5% of their weight at 12-month follow-up and the percentage of weight loss were included as dependent variables (respectively in the logistic and linear regression analysis); baseline clinical parameters and their change from admission to discharge (EDE-Q, IFBS, and WBIS subscales and global scores) were included as independent variables. Age and sex were forced into the model to adjust for their effects.

All statistical analyses were conducted using IBM SPSS Statistics, version 29.0.2.0.

## 3. Results

### 3.1. Sample

A total of 400 eligible patients were included in the study. Their mean age was 55.9 (SD = 14.1) years, and the mean BMI was 41.9 (SD = 8.3) kg/m^2^ (range 30–76.8). The majority (65%) were female.

### 3.2. Attrition and Follow-Up Completion

Three-hundred seventy-one patients completed the treatment, and 29 (7.8%) dropped out. All 371 patients who completed the three weeks of intensive CBT-OB were contacted after 12 months to participate in the follow-up interview. Three-hundred-one patients (81.1%; 301/371) agreed to respond to the telephone follow-up interview, and 70 (18.9%, 70/371) were lost at follow-up (33 refused telephone contact; 36 were not found or provided unreliable data; and 1 was deceased) (Figure 1). No significant differences in demographic or baseline clinical variables were found between patients who participated in the follow-up interview and those who did not.

### 3.3. Response to Treatment

At discharge, patients significantly decreased their body weight and significantly reduced eating disorder psychopathology (except dietary restraint), irrational beliefs about food, and internalized weight stigma (Table 1).

Table 2 shows body weight and BMI at each time point and weight change from baseline to discharge and 12-month follow-up, using completer and intention-to-treat analysis. Two hundred eighty-four out of 301 patients who completed the follow-up interview reported their body weight, so a completer analysis of body weight was conducted on 284 patients.

In completers, RM ANOVA indicated a significant change over time. Pairwise comparisons with post hoc Bonferroni indicated that overall body weight and BMI were significantly higher at admission than at discharge and significantly higher at admission and at discharge than at 12-month follow-up. Similar findings were found for intention-to-treat analysis.

To evaluate the change in binge-eating episodes from baseline to follow-up, the episodes assessed at baseline with the EDE-Q were characterized in the same way as those assessed at the follow-up interview. A significant reduction in binge-eating episodes from admission to follow-up was observed (chi-squared = 145.95, *p* < 0.001) (Table 3).

Finally, considering the questions asked to the patients at the 12-month follow-up interview, 44.2% (133/301) of patients attended post-residential treatment. Among them, 73.7% (98/133) received CBT-OB post-inpatient treatment. Overall, 31.6% (42/133) interrupted post-inpatient treatment, mainly for logistic reasons. Moreover, 65.9% (198/301) reported an improvement in general health status after the inpatient treatment, and more than 35% indicated that they had often, very often, or always felt happy or joyful in the past four weeks. No patient reported taking or having taken weight loss drugs.

### 3.4. Cognitive–Psychological Features as Clinical Predictors of Weight Loss

Hierarchical regression analysis revealed that the factors more closely associated with the percentage of weight change at 12 months were the baseline EDE-Q ‘eating concern’ subscale (beta = −0.24, t = −2.81, *p* = 0.005) and change from admission to discharge in IFBS ‘self-deception on eating and weight control’ (beta = 1.62, t = 2.50, *p* = 0.013), after controlling for age and gender. Similarly, in stepwise logistic regression analysis, the probability of achieving 5% weight loss significantly increased for any point lower in the baseline EDE-Q ‘eating concern’ subscale (OR = 0.59, 95%CI: 0.45–0.78), higher in the baseline EDE-Q ‘weight concern’ subscale (OR = 1.49, 95%CI: 1.08–2.07), and in higher change from admission to discharge in IFBS ‘self-deception on eating and weight control’ (OR = 1.07, 95%CI: 1.01–1.14), independently of age and gender. No other variables were significant in the regression analyses.

## 4. Discussion

This study, evaluating the potential role of cognitive–psychological variables on weight loss in patients with severe obesity treated with intensive residential CBT-OB and re-assessed 12 months after discharge, had three main findings.

### 4.1. Predicting the Role of Cognitive Variables in Weight Loss

The first is that weight loss at the 12-month follow-up was predicted by three psychological variables. In particular, higher change in IFBS ‘self-deception on eating and weight control’ predicted a higher probability of reaching the main outcome of the treatment at the 12-month follow-up. This IFBS factor includes items such as: ‘*You won’t gain weight for anything you eat before 8 p.m.*’; ‘*If I exercise first, I can eat whatever I want*’; ‘*Being overweight is genetic, so why bother trying to lose weight?*’; ‘*Because alcohol has no fat, it can’t make you gain weight*’; ‘*What a person eats really has no effect on their health’*. Higher changes in this variable indicate a higher awareness of the effects of eating on body weight that could contribute to increased weight control in patients with obesity.

Another variable predicting weight loss is eating concern. Lower baseline eating concern predicted a large weight loss. Eating concern EDE-Q subscale includes items such as ‘*Has thinking about food, eating or calories made it very difficult to concentrate on things you are interested in (for example, working, following a conversation, or reading)?*’; ‘*Have you had a definite fear of losing control over eating?*’; ‘*On what proportion of the times that you have eaten have you felt guilty (felt that you’ve done wrong) because of its effect on your shape or weight?*’. Considering the psychological cognitive–behavioral models of eating disorder-maintaining mechanisms [32], we can hypothesize that higher eating concern may induce the patients to adopt dysfunctional dietary restraint, which, through the violation of the control mechanism, promotes dysregulated eating or binge-eating episodes. Lower eating concern could improve flexible restraint and reduce rigid restraint. Some evidence suggests that weight loss success is related to changes in eating behavior traits, with increased flexible restraint and decreased rigid restraint being related to more significant weight loss, and decreased disinhibition predicting weight loss at 12 months [33,34]. In contrast, rigid control of eating behavior was not associated with success during weight loss and its maintenance. In line with these findings, we also found that higher weight concern predicted a large probability of reaching at least 5% weight loss. EDE-Q weight concern includes items such as the following: ‘*Has thinking about shape or weight made it very difficult to concentrate on things you are interested in (for example, working, following a conversation, or reading)?*’; ‘*Have you had a strong desire to lose weight?*’; ‘*Has your weight influenced how you think about (judge) yourself as a person?*’. Maintaining a higher, not pathological, weight concern could allow patients to improve their control of eating. Apparently, in contrast with other studies, we did not find a relationship between baseline internalized weight bias or their change after intensive treatment and weight loss. This discrepancy could be explained by considering the fact that previous studies have evaluated the relationship between weight loss and weight bias internalization as specific for race and gender [17,18]. Moreover, in our study, differently from others [19], we included several cognitive–psychological variables that could have a more powerful effect on weight loss than weight bias internalization.

### 4.2. Treatment Outcome

The second finding of our study is that patients with brief, intensive CBT-OB treatment achieved a substantial and healthy reduction in body weight at the 12-month follow-up. Specifically, the mean weight loss percentage among completers after 12 months was 9.5%. The weight loss percentages were slightly higher than those (8%) reported in a similar time frame by a recent review of lifestyle modification approaches for the treatment of obesity in adults [35].

The third finding from this study is that patients showed a significant reduction in binge-eating episodes, with more than 80% of patients reporting no binge-eating episodes in the last 28 days and at 12 months from discharge, confirming previous findings [36].

### 4.3. Limitations and Strengths

Our study did suffer from certain limitations. The first and main pertains to the self-reported body weight at the 12-month follow-up. This limitation could have produced an overestimation of the lost weight and interfered with our findings [37]. Moreover, the study’s reliance on self-reported measures introduces the possibility of biases, including social desirability and recall bias. Furthermore, the absence of controls for potential confounding variables, such as comorbid mental health conditions, raises concerns about the validity of the findings. Second, the very particular setting, with an initial intensive residential period—rarely used in obesity treatment in the community—makes it difficult to extend the results to the normal outpatient setting. Third, the study overlooks potential cultural and socio-economic factors that may shape irrational food beliefs. Future research should examine how these external influences affect cognitive beliefs and weight loss outcomes across diverse populations. That being said, the study had several strengths. First, to our knowledge, this is the first study investigating a pool of cognitive–psychological variables as potential predictors of weight loss. Second, the treatment was a well-validated intervention delivered in a real-world setting and used some original procedures to personalize the intervention and to address the specific cognitive processes that our previous research has found to be associated with attrition, weight loss, and weight maintenance [10]. Third, the fact that the 12-month follow-up interviews were conducted by expert clinicians not involved in the treatment, rather than relying on self-report questionnaires, enabled us to screen the responses (subjectively) for unreliable data and better investigate and understand the patients’ perspectives.

## 5. Conclusions

In conclusion, our data suggest that weight loss in patients with severe obesity is affected by an early improvement in irrational beliefs (i.e., self-deception on eating and weight control) and in higher basal weight concern. Moreover, lower eating concern independently contributed to weight loss. On the contrary, the other irrational beliefs about food, the internalized weight stigma, restraint, and shape concern did not predict weight loss. If confirmed, these findings suggest also focusing the interventions of weight loss on addressing specific cognitive–psychological processes potentially implicated in weight loss success. In particular, weight loss interventions could include cognitive–behavioral strategies to address irrational beliefs and consider that patients with lower baseline weight concern and higher eating concern could obtain worse outcomes from the weight loss treatment.

## Figures and Tables

**Table 1:** Admission and discharge clinical characteristics of 371 patients with obesity who completed the intensive inpatient treatment at Villa Garda Hospital. Data are presented as mean (SD) or number (%).

Completers Analysis	Admission	Discharge	*T*-Test for Paired Samples	*p*-Value
Body weight, kg	115.9 (27.2)	111.1 (25.3)	33.69	<0.001
Body Mass Index, kg/m^2^	41.9 (8.3)	40.3 (7.8)	37.09	<0.001
Eating Disorder Examination Questionnaire, mean (SD)				
- Global score	2.55 (1.1)	2.12 (1.1)	8.60	<0.001
- Dietary restraint	1.60 (1.3)	1.66 (1.4)	0.62	0.532
- Eating concern	1.81 (1.6)	1.15 (1.2)	8.83	<0.001
- Weight concern	3.15 (1.3)	2.59 (1.3)	9.05	<0.001
- Shape concern	3.64 (1.5)	3.09 (1.6)	8.88	<0.001
Irrational food beliefs scale, mean (SD)				
- Self-deception on eating and weight control	21.89 (5.8)	21.17 (6.0)	2.32	0.021
- Beliefs about eating and emotion regulation	18.93 (5.1)	17.18 (4.9)	7.42	<0.001
- Low tolerance for eating control	17.28 (4.7)	15.53 (4.4)	8.23	<0.001
- Beliefs about eating and hedonic pleasure	8.26 (2.5)	7.77 (2.4)	3.89	<0.001
- Beliefs about dieting	4.67 (1.6)	4.42 (1.5)	2.51	0.013
- All-or-nothing thinking about eating	9.97 (2.5)	9.05 (2.4)	6.27	<0.001
Weight bias internalization stigma, mean (SD)	3.79 (1.2)	3.42 (1.3)	6.56	<0.001

**Table 2:** Body mass index, weight change, and 5-10% weight change from baseline to 12-month follow-up in patients with obesity treated with CBT-OB. Data from completer and intention-to-treat analyses. For repeated measure (RM) ANOVA: Mauchly’s sphericity tests were >0.05.

**Completer Analysis (n = 284)**				
	**Admission**	**Discharge**	**12-Month Follow-Up**	**RM ANOVA (F; *p*-Value)**
Body weight in kg, mean (SD)	114.6 (26.9)	109.9 (24.9)	103.4 (25.0)	F = 292.57, *p* < 0.001
Body mass index in kg/m^2^, mean (SD)	41.6 (8.1)	39.9 (7.6)	37.6 (7.8)	F = 304.89; *p* < 0.001
Weight loss from admission in kg, mean (SD)	--	4.6 (2.8)	11.2 (11.0)	---
Mean percentage weight loss from admission	--	3.9 (1.6)	9.5 (8.5)	---
≥5% weight loss from admission, n (%)	--	70 (24.6%)	191 (63.3%)	---
**Intention-to-Treat Analysis (n = 400)**				
	**Admission**	**Discharge**	**12-Month Follow-Up**	**RM ANOVA**
				**Time**
Body weight in kg, mean (SD)	115.8 (1.4)	110.9 (1.3)	105.8 (1.6)	F = 93.51, *p* < 0.001
Body mass index in kg/m^2^, mean (SD)	41.9 (0.4)	40.2 (0.4)	38.4 (0.5)	F = 63.39; *p* < 0.001
Weight loss from admission in kg, mean (SD)	--	4.8 (0.5)	9.9 (1.4)	---
Mean percentage weight loss from admission	--	3.8 (0.4)	7.5 (1.2)	---
≥5% weight loss from admission, n (%)	--	109 (27.2%)	248 (62.0%)	---

**Table 3:** Binge-eating episodes at admission and at 12-month follow-up in 301 patients with severe obesity who completed the follow-up interview. Data are presented as frequency and percentage.

	Admission	12-Month Follow-Up	Chi-Squared Test; *p*-Value
Objective binge-eating episodes, n (%) - No episodes - Fewer than 1 episode per week - 1 episode per week - 2–3 episodes per week - 4–7 episodes per week - 8–13 episodes per week - 14 or more episodes per week	238 (79.1%) 12 (4.0%) 11 (3.7%) 19 (6.3%) 16 (5.3%) 2 (0.7%) 3 (1.0%)	247 (82.0%) 8 (2.7%) 11 (3.6%) 21 (7.0%) 8 (2.7%) 1 (0.3%) 5 (1.7%)	145.95; <0.001

## Data Availability

The data presented in this study are available from the corresponding author upon request due to ethical restrictions.

## Abbreviations

The following abbreviations are used in this manuscript:

BMI	Body Mass Index
CBT-OB	Cognitive–Behavioral Therapy for Obesity
EDE-Q	Eating Disorder Examination Questionnaire
IFBS	Irrational Food Beliefs Scale
WBIS	Weight Bias Internalization Scale
